# Genetic and pathological findings in a boy with psoriasis and C3 glomerulonephritis: A case report and literature review

**DOI:** 10.1002/mgg3.1430

**Published:** 2020-07-28

**Authors:** Lei Wei, Ye Fang, Guanghai Cao, Shufeng Zhang, Ming Tian, Qian Shen, Hong Xu, Cuihua Liu, Jia Rao

**Affiliations:** ^1^ Department of Nephrology and Rheumatology Children's Hospital of Zhengzhou University Henan Children’s Hospital Zhengzhou Children’s Hospital Zhengzhou Henan China; ^2^ Department of Nephrology Children's Hospital of Fudan University National Pediatric Medical Center of China Shanghai China; ^3^ Shanghai Kidney Development and Pediatric Kidney Disease Research Center Shanghai China; ^4^ Shanghai Key Lab of Birth Defect Children's Hospital of Fudan University Shanghai China

**Keywords:** *CARD14*, C3 glomerulonephritis, *CLCN5*, Dent disease, proteinuria, psoriasis

## Abstract

**Background:**

Psoriasis is a chronic inflammatory dermatosis with complex genetic basis supported by family investigation. Renal involvement in psoriasis is sparsely studied and its pathogenesis is still unclear.

**Methods and Results:**

We describe the case of a 7‐year‐old boy presented new onset of nephropathy two weeks after a flare‐up of psoriasis. His mother had a long history of psoriasis without abnormal urinalysis records. The case showed non‐nephrotic range proteinuria, microscopic hematuria without any other abnormal results including renal function, complement cascade, and ultrasound. Renal pathological demonstrated the diagnosis of C3 glomerulonephritis (C3GN) showing mesangial proliferative glomerulonephritis with C3 staining only, effacement of podocyte process and intramembranous electron dense deposit by electric microscopy. Parent‐child trio WES performed to screening the common variants of psoriasis susceptibility locus and also the rare variants associated with C3GN. We identified a missense single nucleotide polymorphism of *CARD14* (*607211, rs34367357, p.Val585Ile) carried by the proband and his mother. Meta‐analysis proved the association of rs34367357 and psoriasis (*p* = 0.006, OR = 1.23). A hemizygouse mutation of *CLCN5* (*300008, c.1904A＞G,p.Asn635Ser) was identified for diagnosis of Dent disease (*300009).

**Conclusion:**

The case highlights the genetic study is necessary to facilitate disease differentiation in new onset of nephropathy with psoriasis in children.

## Graphical abstract

A case of a 7‐year old boy presented new onset of nephropathy two weeks after a flare‐up of psoriasis. Pathological findings and genetic study identified the diagnosis of C3 glomerulonephritis with psoriasis accompanied by Dent disease.

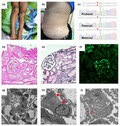

## 1 BACKGROUND

Psoriasis is a hereditary, chronic inflammatory disorder of the skin with a strong genetic background (Boehncke & Schon, 2015). The psoriatic process is causally associated with systemic diseases such as cardiovascular diseases, diabetes mellitus and renal damage. Although renal involvement is not common during the course of psoriasis, certain glomerular diseases including secondary renal amyloidosis, IgA nephropathy, membranous glomerulopathy and membranoproliferative glomerulonephritis have been reported in psoriatic patients (Boehncke & Schon, 2015; Liu et al., 2018). In this paper, we describe a patient developed C3 nephropathy during flare of psoriasis who was further diagnosed with *CLCN5* (*300008) mutation for diagnosis of Dent disease (*300009).

## 2 CASE PRESENTATION

A 7‐year‐old Chinese boy came to consult with persistent proteinuria and microscopic hematuria two weeks post the fare‐up of psoriatic skin disease with no history of renal disease.

**Figure 1:**
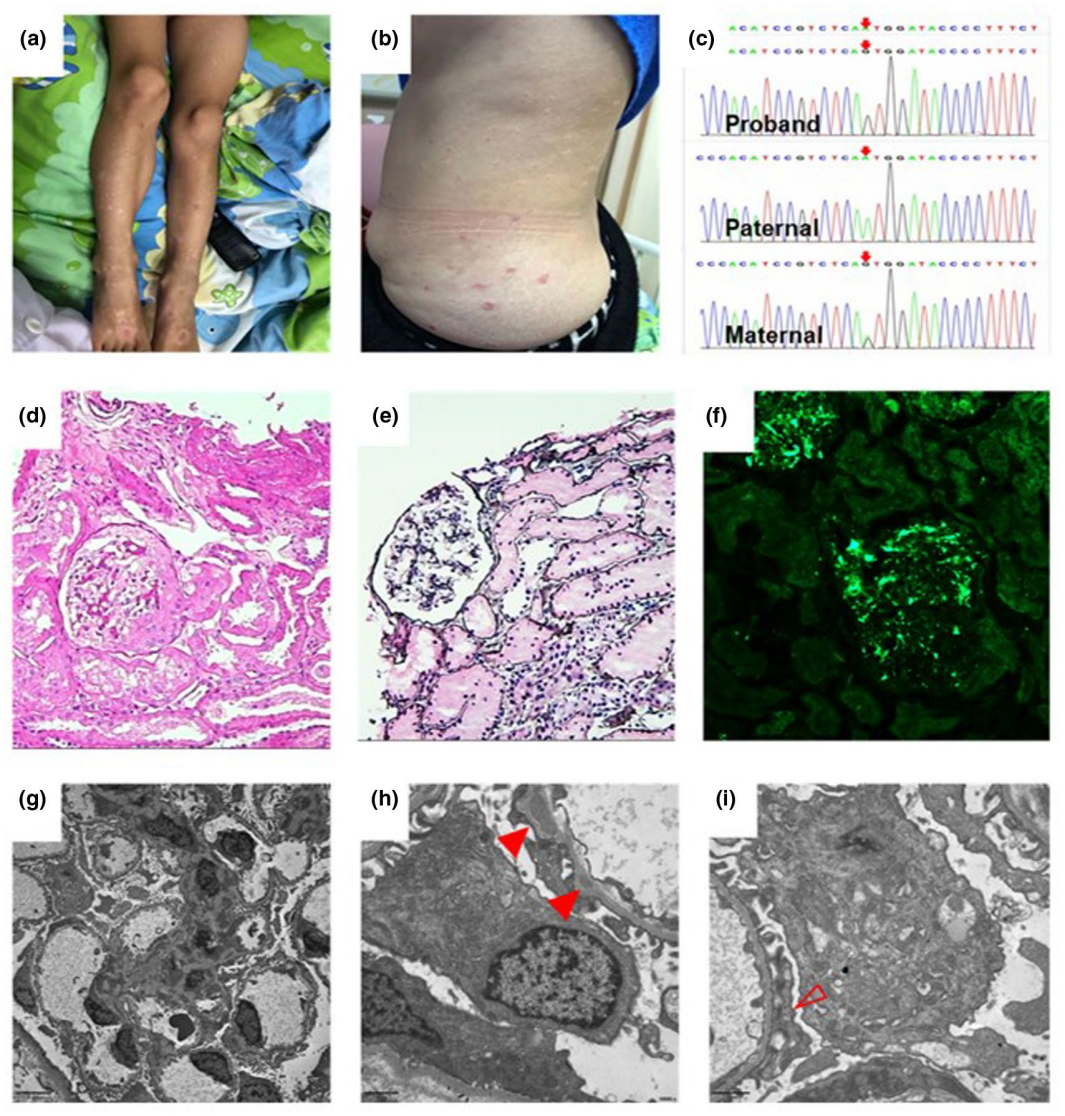
(a) Clinical presentation of the proband erythrodermic psoriasis. Patient develop multiple erythematous, hyperkeratotic plaques, and papules with silvery squamous of psoriasis on lower extremities. (b) The skin lesions on the trunk of the mother of the proband. (c) Gene sequencing of a mutation of *CLCN5* gene located in X chromosome: c.1904A>G, p.Asn635Ser (proband, hemi; paternal, wt; maternal, het). (d and e) Light microscopy findings show the mesangial proliferation glomerulonephritis. (f) Immunofluorescence shows bright staining of C3 in the mesangial region. (g–i) Electron microscopy findings show intramembranous electron dense deposits (solid triangle) and podocyte foot process infusion (hollow triangle)

He was the first child of a non‐consanguineous Chinese couple. He was born at term via cesarean section without any notable abnormalities. His birth weight was 3500 g (50–90th centile). He was diagnosed with new onset of psoriasis and received topical medication at a local hospital two weeks ago. No oral steroid therapy, methotrexate, cyclosporin A, or nonsteroidal anti‐inflammatory drugs were used since the diagnosis was made. His mother had a long history of psoriasis without other systemic damage or abnormal urinalysis recodes. His father and younger brother were in good health. There was no other considerable morbidity in her medical history. The patient gave no history of the passage of clots, burning urination, hesitancy, or precipitancy of urination. There was no history of oliguria, pedal edema, facial puffiness, joint pain, fever, score throat, or recent vaccination. Upon admission, the patient's vital signs were within normal limits with height of 128 cm and a blood pressure reading of 95/50 mmHg. Upon physical examination, multiple erythematous, hyperkeratotic plaques, and papules with silvery squamous were observed on both upper and lower extremities (Figure 1A). Examination of all the other systems including version and hearing test revealed no abnormality.

His urinalysis showed red blood cells of 20‐50/HP and moderate level of proteinuria. A 24‐h urine collection revealed 265–364 mg of protein. Urine for dysmorphic RBC was negative (2%). Serial detection of urinary microprotein showed the raise of low‐molecular‐weight protein fraction with α1 microglobulin of 2.93 mg/L (Ref. <12 mg/L), microalbumin of 626 mg/L (Ref. <30 mg/L), β2 microglobulin of 537.4 µg/L (Ref. <300 µg/L). However, the urine protein electrophoresis revealed the raise of mainly albumin and transferrin without notable bands of α1 microglobulin, α2 macroglobulin, β2 microglobulin, or gamma globulins (IgG/IgA/IgM) which indicated the glomerular proteinuria. The urine calcium creatinine ratio was 0.51, which was slightly higher than that of patients in the same age group. While, a 24‐h urine collection revealed only 0.06 mmol/kg of calcium. The inflammatory markers were mildly abnormal with a CRP <8 mg/dl, ESR 21 mm/h (0‐15 mm/h) and antistreptolysin O titer (ASO) 315.63 (<200 IU/ml). Serum creatinine was 43 ummol/L, albumin 42.1 g/L, C3 and C4 were 1.03 (0.67–1.76 g/L) and 0.11 (0.1–0.4 g/L), respectively. Coagulation profile was normal. Serologically he had negative ANA, pANCA/cANCA, and dsDNA. Ultrasonography showed normal size of kidneys with maintained cortical medullary differentiation.

In view of persistent microscopic hematuria and probable glomerular etiology, the patient was advised for renal biopsy. Our provisional diagnosis was IgA nephropathy. However, kidney biopsy revealed mesangial proliferation glomerulonephritis with C3 staining only (Figure 1d–f). No observations of glomerular sclerosis or segmental sclerosis were shown. Immunofluorescence indicated dominant mesangial and capillary wall staining for C3 with slightly IgM deposit and no staining for IgG, IgA, C1q. Electron microscopy revealed segmental fusion of the glomerular foot process and an amount of electronic dense deposits in the mesangial region and intramembranous region (Figure 1g–i). It indicated the glomerular basement thickness varying from 263.1 nm to 519.8 nm ruling out thin basement membrane disease. Further observation of podocyte subcellular organelles did not reveal any significant abnormalities. Thus, confirming a diagnosis of C3 glomerulonephritis (C3GN). Afterward, serum investigation into the complement cascade was performed and the level of C3, C4, and C3 nephritic factor came to be normal.

## 3 GENETIC ANALYSIS

In order further identify and confirm the diagnosis, we performed the trio whole exon sequencing (Trio‐WES) including proband and parents sequenced concurrently after the informed consent was obtained from his parents. Samples were subjected to WES in outsourcing, and raw data were transferred to our lab for the bio‐informatic analysis. The genetic approach to WES followed the Mendelian disease initially by analyzing the rare variants combined with phenotype and then screening the common variants of single nucleotide polymorphism (SNPs) in candidate genes of psoriasis susceptibility locus (PSORS) and risk alleles of C3GN (Table S1).

After profound discussion and review with the consults of national multicenter registry (Chinese Children Genetic Kidney Disease Database, CCGKDD) (Rao et al., 2019), it was confirmed that the boy carried a mutation (c.1904A>G, p.Asn635Ser) of *CLCN5* (*300008) gene located in X chromosome. Pedigree loci found that his mother was heterozygous while his father was wild‐type whose clinical phenotype was normal (Figure 1C). The rare mutation is not reported in the database gnomAD (https://gnomad.broadinstitute.org/). The possible impact on the amino acid substitutions on the structure and function of *CLCN5* was assessed using four bioinformatics tools: SIFT showed prediction of Damaging (https://sift.bii.a‐star.edu.sg/sift‐bin/), PolyPhen‐2 score indicated 0.99 (http://genetics.bwh.harvard.edu/cgi‐bin/pph2), MutationTaster showed prediction of Disease causing with score of 0.999 (http://www.mutationtaster.org/cgi‐bin/). The boy was therefore diagnosed as type I Dent disease with incomplete phenotype.

We performed the further screening of the common variants in the candidate genes associated psoriasis or C3GN by using of the digital panel including 119 genes (Table S1). Among variants shared by the two affected individuals and not present in the unaffected father and younger brother, the known SNPs associated with psoriasis in CARD14 (*607211*)* gene resulted worth of further investigation. In particular, it was a common SNP (rs34367357) of an exon 12 heterozygous nucleotide change, c.1753G>A, leading to the missense amino acid substitution p.Val585Ile (p.V585I). The presence of this variant was validated by Sanger sequence in the affected members who underwent WES, and in the rest of the available family members. We didn't confirm any rare variants or risk alleles in candidate genes associated with C3GN or complement cascade deficiency.

## 4 LITERATURE REVIEW FOR *CARD14* AND PSORIASIS

**Figure 2:**
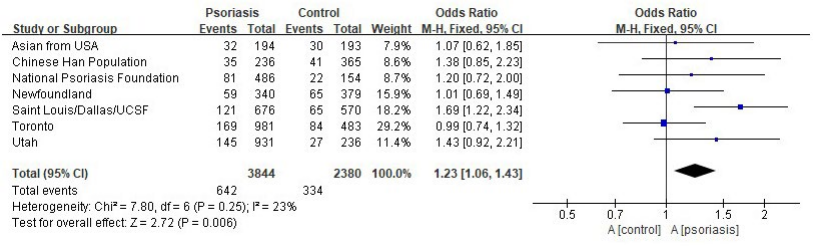
Test for association of Psoriasis with rs34367357 in *CARD14* across 7 case/control cohorts. Fixed‐effects meta‐analysis for rs34367357 were shown. Forest plots indicate the direction of effect, relative weight, and confidence interval for the odds ratio of this SNP in each cohort. The number of cases and controls successfully genotyped in each cohort is shown, and the meta‐analysis OR and *p* value are listed below each plot. Cohorts of National Psoriasis Foundation/Newfoundland/St. Louis/Dallas/UCSF/Toronto/Utah are independent case/control cohorts of Northern European ancestry (International Psoriasis Genetics, 2003; Israel & Mellett, 2018; Jordan, Cao, Roberson, Duan, et al., 2012; Jordan, Cao, Roberson, Pierson, et al., 2012). Cohort of Asian are from USA includes samples of Asian ancestry from the St. Louis/Dallas/UCSF (University of California, San Francisco) and the National Psoriasis Foundation Victor Henschel Tissue Repository cohorts (Jordan, Cao, Roberson, Duan, et al., 2012). Cohort of Chinese Han population are from CHINA (Qin et al., 2014). CI, confidence interval; OR, odds ratio; SNP, single nucleotide polymorphism

Genome‐wide linkage analysis studies identified nine “Psoriasis Susceptibility” regions or loci (*PSORS1*‐*9*: **#**177900, #602723, **%**601454, **%**603935, **%** 604316, **%** 605364, **%**10706, **%**610707, **%**607857) including identification of *CARD14* as being the responsible gene for the underlying association of the *PSORS2* locus with psoriasis. We conducted a literature search of PubMed and Cochrane database with the search terms “psoriasis,” “genetic,” and “*CARD14*” (International Psoriasis Genetics, 2003; Israel & Mellett, 2018; Jordan, Cao, Roberson, Duan, et al., 2012; Jordan, Cao, Roberson, Pierson, et al., 2012; Scudiero et al., 2011). A meta‐analysis of the SNP (rs34367357) was performed in the seven case/control cohorts of psoriasis. It revealed the further evidence of association between psoriasis and rs34367357 (fixed effect *p* value = 0.006; OR = 1.23 [1.06, 1.43]) (Figure 2, Table S2).

## 5 TREATMENT AND FOLLOW‐UP

The boy was started on captopril daily with regularly follow‐up of urine analysis and renal function. After starting therapy, in the past twelve months, there has been no episode of gross hematuria and patient has been doing well. Microscopic hematuria and proteinuria persisted without renal insufficiency. During the episodes of respiratory infection, his urinalysis revealed the raise of red blood cells (321/µl) and 24‐h urine protein excretion was only 273 mg/day. The clinical records showed the urine β2 microglobulin of 498.9 µg/L (ref. <300 µg/L), and urine microalbumin of 397 mg/L (ref. <30 mg/L). The skin lesions of psoriasis remained stable without additional medicine.

## 6 DISCUSSION

Psoriasis is a common chronic inflammatory skin disease with a complex genetic background affecting approximately 1%–2% of the world's population (Boehncke & Schon, 2015). Renal involvement in psoriasis has been demonstrated by several reports (Visconti et al., 2016). The presentation of these patients may range from asymptomatic proteinuria and hematuria to clinically significant generalized edema, hypertension, and high levels of proteinuria accompanied by elevated creatinine and urea levels (Boehncke & Schon, 2015; Visconti et al., 2016). Various studies have been undertaken in an effort to clarify the pathogenetic mechanisms of renal damage associated with psoriasis. They commonly emphasized the role of underlying genetic and immunologic mechanisms. IgA nephropathy has been the most common findings in renal biopsy specimens (as a mesangioproliferative type) in psoriatic patients. Amyloidosis, membranous nephropathy, and focal proliferative glomerulopathy were also reported to occur in psoriatic patients (Afonina et al., 2016; Boehncke & Schon, 2015; Israel & Mellett, 2018; Visconti et al., 2016). Drug‐induced nephrotoxicity probably depends on dose‐related toxicity of nonsteroidal anti‐inflammatory drugs and interstitial fibrosis, tubular atrophy, and glomerulosclerosis due to cyclosporine A (Israel & Mellett, 2018; Visconti et al., 2016). Clinical presentation of C3 glomerulopathy with psoriasis is extremely rare. Only one case report from India described C3GN in a psoriatic young man without any genetic information (Balwani, Pasari, & Tolani, 2019).

We reported a pediatric case of C3GN associated with psoriasis accompanied by Dent disease. C3GN is a disease entity that has been identified in patients with acquired abnormalities or genetic mutations (Barbour, Pickering, & Cook, 2013), including complement H factor or complement I factor, which was identified normal in our patient. The renal pathological findings in this case of active signs of psoriasis showed mesangioproliferative glomerulonephritis with C3 deposits. We postulate the uncontrolled inflammatory process of psoriasis may lead to dysregulated alternative pathway activation. Considering the relapse of hematuria during the episode of respiratory infection, the triggering event of infection got special attention. Infection can active the alternative complement pathway and uncover a genetic predisposition to C3GN. It has been reported that the 28.9% cases presented post infection in the cohort study of C3GN (Schena, Esposito, & Rossini, 2020). From a pathology standpoint, there was intramembranous and mesangial C3 deposits with the absence of subepithelial humps. It helps differentiate this case of C3GN from the infection‐related glomerulonephritis. Other possibilities of infection‐related glomerulonephritis like HIV and HCV related nephropathies were shown negative in this case. Furthermore, genetic study for this case was performed to screening for the mutations related to complement cascade.

Based on the family history of psoriasis, genetic work for this case was carried out focusing on the *PSORS*, including *CARD14* (Caspase Recruitment Domain‐containing protein 14, *607211) (also called *CARMA2* or *BIMP2*). CARD14 recruits interacting partners BCL10 and MALT1 to form the so‐called CBM complex and thus initiate NF‐κB and MAPK signaling pathway (Liu et al., 2018; Scudiero et al., 2011). BCL10 and MALT1 are ubiquitously expressed proteins whereas the CARD molecule gives cell specificity to the complex, with CARD14 localizing in skin and mucosal tissues (Scudiero et al., 2011; Visconti et al., 2016). CARD14 was discovered to be highly expressed in keratinocytes and was identified as the causative gene at the *PSORS 2* locus, which had previously been identified as one of the principle risk loci for psoriasis (International Psoriasis Genetics, 2003; Israel & Mellett, 2018)^.^ Autosomal dominant gain of function (GoF) mutations in *CARD14* is associated with various entities in the psoriasis disease spectrum. By screening seven psoriasis cohorts with varying ancestries (over 6000 cases and 4000 controls), it has been identified 15 additional rare and common *CARD14* variants that were enriched in cases over controls (International Psoriasis Genetics, 2003; Jordan, Cao, Roberson, Duan, et al., 2012). There was strong evidence of GoF variants based on their ability to trigger NF‐κB activity and an inflammatory gene signature (International Psoriasis Genetics, 2003; Jordan, Cao, Roberson, Pierson, et al., 2012). Heterozygous mice harboring a GoF mutation in the *Card14* (*Card14*ΔE138) spontaneously developed a chronic psoriatic skin disease at 5 days‐old (Mellett et al., 2018). Thus, *CARD14* harbors rare and highly‐penetrant mutations that segregate in multiplex pedigrees, as well as common susceptibility alleles of small effect. Here we found a SNP (rs34367357) of *CARD14* carried by the proband and his mother with psoriasis after screening the candidate genes through WES. We identified significant associations with the SNPs (rs34367357) and psoriasis in the European ancestry and Asians. The function of the SNPs (rs34367357) remains unknown. Delineating the correlation between genotype of *CARD14* and phenotype of psoriasis could contribute to our understanding of the pathogenesis of psoriasis.

Interestingly we also identified a hemizygous mutation in *CLCN5* in the male proband with a diagnosis of Dent disease. To our knowledge, there is no report on comorbidity of Dent disease and C3GN. Dent disease (*300009) is a rare X‐link renal proximal tubular disorder characterized by low‐molecular‐weight proteinuria (LMWP), hypercalciuria, nephrocalcinosis, urolithiasis, and eventually progression to end stage renal failure (Claverie‐Martin, Ramos‐Trujillo, & Garcia‐Nieto, 2011). The incidence of hypercalciuria is as high as 92% in European and American patients whereas data from Asian shows only 51% (Blanchard et al., 2016; Claverie‐Martin et al., 2011; Takemura et al., 2001). Dent disease is a hereditary renal tubular disease as well as a podocyte disease. More than half of the patients with Dent disease have nephrotic range of proteinuria (Claverie‐Martin et al., 2011; Takemura et al., 2001; Ye et al., 2020). The glomerular pathological damage is as common as tubulointerstitial damage. The findings of glomerular sclerosis in almost two thirds of the biopsies showed about 10% glomeruli being sclerosed. And 75% cases reported the fusion of podocyte foot process in glomeruli (Blanchard et al., 2016; Wang et al., 2016). *CLCN5* knockdown in podocytes resulted in defective transferrin endocytosis and other podocyte function deficiency (Solanki et al., 2018). In the present patient, no sclerosing glomeruli or notable tubulopathy were found. While the podocyte damage was indicated by foot process fusion and intramembranous dense deposits under electron microscopy. Clinically, analysis of urine protein showed the predominant glomerular proteinuria without hypercalciuria. Taken together, Dent disease should be taken into consideration with the missense mutation in *CLCN5* with incomplete phenotype.

Taking into consideration the above, the working diagnosis in this case was that of C3GN associated with psoriasis and comorbidity of Dent disease. As shown by the recommendations obtained from case series and observational studies, children and adults with C3GN in presence of mild or moderate proteinuria should receive supportive therapy with angiotensin‐converting enzyme inhibitors (ACEI) or angiotensin receptor blockers (Barbour et al., 2013; Schena et al., 2020). The treatment strategy, in the present case, targeted reducing proteinuria and preserve renal function with ACEI drugs. Long‐term follow‐up of proteinuria and renal function should be taken carefully.

## 7 CONCLUSION

Psoriatic patients should be followed up by close monitoring of urinalysis and renal functions for early diagnosis and treatment of coexisting glomerular lesions. Genetic study in children with renal involvement and psoriasis could facilitate disease differentiation in new onset of nephropathy with psoriasis and spare the patients from aggressive therapy.

## CONFLICT OF INTERESTS

The authors declare that they have no competing interest.

## AUTHORS’ CONTRIBUTIONS

LW and YF contributed to acquisition of data, draft, and wrote the paper. JR, GHC, SFZ, MT, QS, and CHL contributed to interpretation of data, concept, and revision. JR contributed to study concept and design. HX, JR, and CHL contributed to revision. All authors have read and approve the final version of manuscript.

## ETHICS APPROVAL AND CONSENT TO PARTICIPATE

All procedure performed in studies involving human participants were in accordance with the ethical standards of the Ethical Committee for scientific research approval of the Children's Hospital of Fudan university (NO. 2018_286). Informed consent Written informed consent was obtained from all patients and their parents for publication.

## CONSENT FOR PUBLICATION

Written consent for publication was obtained from person presented in this case report.

## Supporting information

Table S1‐S2
