# Supplementary material for: Genetic and pathological findings in a boy with psoriasis and C3 glomerulonephritis: A case report and literature review
Source: Mol Genet Genomic Med. 2020 Jul 28;8(10):e1430. doi: 10.1002/mgg3.1430 (PMC7549556; doi:10.1002/mgg3.1430)
Supplement: Supplementary file 1 — Table S1‐S2 [file MGG3-8-e1430-s001.docx]

**Supplementary Table 1. 119 candidate genes associated with phenotype of psoriasis.**

| AP1S3 | Adaptor-related protein complex 1, sigma 3 subunit |
| --- | --- |
| ARVCF | Armadillo repeat gene deleted in velocardiofacial syndrome |
| BRAF | V-raf murine sarcoma viral oncogene homolog B |
| BTD | Biotinidase |
| C1orf172 | Chromosome 1 open reading frame 172 |
| C5 | Complement component 5 |
| CACNA1G | Calcium channel, voltage-dependent, T type, alpha 1G subunit |
| CARD11 | Caspase recruitment domain family, member 11 |
| CARD14 | Caspase recruitment domain family, member 14 |
| CASP8 | Caspase 8, apoptosis-related cysteine peptidase |
| CASR | Calcium-sensing receptor |
| CCL11 | Chemokine (C-C motif) ligand 11 |
| CCL3 | Chemokine (C-C motif) ligand 3 |
| CCL3L1 | Chemokine (C-C motif) ligand 3-like 1 |
| CD209 | CD209 molecule |
| CD28 | CD28 molecule |
| CD3G | CD3g molecule, gamma (CD3-TCR complex) |
| COMT | Catechol-O-methyltransferase |
| CSTA | Cystatin A (stefin A) |
| CTLA4 | Cytotoxic T-lymphocyte-associated protein 4 |
| CX3CR1 | Chemokine (C-X3-C motif) receptor 1 |
| CXCL12 | Chemokine (C-X-C motif) ligand 12 |
| CXCR1 | Chemokine (C-X-C motif) receptor 1 |
| CYBA | Cytochrome b-245, alpha polypeptide |
| CYBB | Cytochrome b-245, beta polypeptide |
| DHCR7 | 7-dehydrocholesterol reductase |
| DNAJC21 | DnaJ (Hsp40) homolog, subfamily C, member 21 |
| DOCK8 | Dedicator of cytokinesis 8 |
| DSG1 | Desmoglein 1 |
| EDA | Ectodysplasin A |
| EDAR | Ectodysplasin A receptor |
| EDARADD | EDAR-associated death domain |
| EFTUD1 | Elongation factor Tu GTP binding domain containing 1 |
| FECH | Ferrochelatase |
| FERMT1 | Fermitin family member 1 |
| FLG | Filaggrin |
| FLI1 | Fli-1 proto-oncogene, ETS transcription factor |
| FOXP3 | Forkhead box P3 |
| GATA3 | GATA binding protein 3 |
| GINS1 | GINS complex subunit 1 (Psf1 homolog) |
| GNA11 | Guanine nucleotide binding protein (G protein), alpha 11 (Gq class) |
| GP1BB | Glycoprotein Ib (platelet), beta polypeptide |
| HDAC4 | Histone deacetylase 4 |
| HIRA | Histone cell cycle regulator |
| HLA-C | Major histocompatibility complex, class I, C |
| HLA-DQB1 | Major histocompatibility complex, class II, DQ beta 1 |
| HLA-DRB1 | Major histocompatibility complex, class II, DR beta 1 |
| HLCS | Holocarboxylase synthetase (biotin-(proprionyl-CoA-carboxylase (ATP-hydrolysing)) ligase) |
| HPGD | Hydroxyprostaglandin dehydrogenase 15-(NAD) |
| HSPA9 | Heat shock 70kDa protein 9 (mortalin) |
| IFIH1 | Interferon induced with helicase C domain 1 |
| IFNG | Interferon, gamma |
| IL10 | Interleukin 10 |
| IL23R | Interleukin 23 receptor |
| IL2RA | Interleukin 2 receptor, alpha |
| IL36RN | Interleukin 36 receptor antagonist |
| IL4R | Interleukin 4 receptor |
| IL7R | Interleukin 7 receptor |
| JMJD1C | Jumonji domain containing 1C |
| KANSL1 | KAT8 regulatory NSL complex subunit 1 |
| KIR3DL1 | Killer cell immunoglobulin-like receptor, three domains, long cytoplasmic tail, 1 |
| KRT1 | Keratin 1 |
| KRT16 | Keratin 16 |
| KRT9 | Keratin 9 |
| LBR | Lamin B receptor |
| LIG4 | Ligase IV, DNA, ATP-dependent |
| MBTPS2 | Membrane-bound transcription factor peptidase, site 2 |
| MCCC2 | Methylcrotonoyl-CoA carboxylase 2 (beta) |
| MSMO1 | Methylsterol monooxygenase 1 |
| MSN | Moesin |
| NCF1 | Neutrophil cytosolic factor 1 |
| NCF2 | Neutrophil cytosolic factor 2 |
| NCF4 | Neutrophil cytosolic factor 4, 40kDa |
| NDNL2 | Necdin-like 2 |
| NEK9 | NIMA-related kinase 9 |
| NFKB2 | Nuclear factor of kappa light polypeptide gene enhancer in B-cells 2 (p49/p100) |
| NOD2 | Nucleotide-binding oligomerization domain containing 2 |
| NSUN2 | NOP2/Sun RNA methyltransferase family, member 2 |
| PAH | Phenylalanine hydroxylase |
| PCCA | Propionyl CoA carboxylase, alpha polypeptide |
| PCCB | Propionyl CoA carboxylase, beta polypeptide |
| PGM3 | Phosphoglucomutase 3 |
| PIGA | Phosphatidylinositol glycan anchor biosynthesis, class A |
| PIK3CA | Phosphatidylinositol-4,5-bisphosphate 3-kinase, catalytic subunit alpha |
| PTPRC | Protein tyrosine phosphatase, receptor type, C |
| RBCK1 | RanBP-type and C3HC4-type zinc finger containing 1 |
| RBM8A | RNA binding motif protein 8A |
| RNU4ATAC | RNA, U4atac small nuclear (U12-dependent splicing) |
| RREB1 | Ras responsive element binding protein 1 |
| SBDS | Shwachman-Bodian-Diamond syndrome |
| SEC24C | SEC24 family member C |
| SHOC2 | Soc-2 suppressor of clear homolog (C. elegans) |
| SLC29A3 | Solute carrier family 29 (equilibrative nucleoside transporter), member 3 |
| SLC30A2 | Solute carrier family 30 (zinc transporter), member 2 |
| SLCO2A1 | Solute carrier organic anion transporter family, member 2A1 |
| SMARCA2 | SWI/SNF related, matrix associated, actin dependent regulator of chromatin, subfamily a, member 2 |
| SPINK5 | Serine peptidase inhibitor, Kazal type 5 |
| SRD5A3 | Steroid 5 alpha-reductase 3 |
| SRP54 | Signal recognition particle 54kDa |
| STAT1 | Signal transducer and activator of transcription 1, 91kDa |
| STAT3 | Signal transducer and activator of transcription 3 (acute-phase response factor) |
| SUOX | Sulfite oxidase |
| TAF1 | TAF1 RNA polymerase II, TATA box binding protein (TBP)-associated factor, 250kDa |
| TBX1 | T-box 1 |
| TGM5 | Transglutaminase 5 |
| TLR3 | Toll-like receptor 3 |
| TMC6 | Transmembrane channel-like 6 |
| TMC8 | Transmembrane channel-like 8 |
| TNFRSF1B | Tumor necrosis factor receptor superfamily, member 1B |
| TP63 | Tumor protein p63 |
| TRAF3IP2 | TRAF3 interacting protein 2 |
| TRAF6 | TNF receptor-associated factor 6, E3 ubiquitin protein ligase |
| TRPM1 | Transient receptor potential cation channel, subfamily M, member 1 |
| TTC7A | Tetratricopeptide repeat domain 7A |
| UFD1L | Ubiquitin fusion degradation 1 like (yeast) |
| WAS | Wiskott-Aldrich syndrome |
| WIPF1 | WAS/WASL interacting protein family, member 1 |
| ZAP70 | Zeta-chain (TCR) associated protein kinase 70kDa |
| ZNF750 | Zinc finger protein 750 |

**Supplementary Table 2. Psoriasis Cases and Controls Included in this Study**

| Cohort Description | Cases | Controls |
| --- | --- | --- |
| Asian from USA | 194 | 193 |
| National Psoriasis Foundation | 486 | 154 |
| Newfoundland | 340 | 379 |
| Chinese Han Population | 236 | 365 |
| Saint Louis/Dallas/UCSF | 676 | 570 |
| Toronto | 981 | 483 |
| Utah | 931 | 236 |

- Cohort of Asian from USA were recruited from the Cardiovascular Research Institute and Center for Human Genetics at the University of California, San Francisco, from the University of Toronto and Toronto Western Hospital and the Department of Medicine, or from the NPF. (Jordan, et al. 2012)
- Cohort of National Psoriasis Foundation samples were from the National Psoriasis Foundation Victor Henschel Tissue Repository (NPF).(Jordan, et al. 2012)
- Cohort Newfoundland were gathered from the Department of Medicine, Division of Rheumatology, Memorial University of Newfoundland.
- Cohort of Chinese Han Population were from Shandong province, CHINA.(Qin, et al. 2014)
- Cohort of Saint Louis/Dallas/UCSF were recruited from either Washington University in St. Louis or the Department of Dermatology at the University of California, San Francisco (UCSF).Controls of A were unaffected individuals who were over 20 years of age and who had no family history of psoriasis; they were recruited from the Texas Scottish Rite Hospital for Children or from the Cardiovascular Research Institute and Center for Human Genetics at the University of California, San Francisco or they were CEU (Utah residents with Northern and Western European ancestry from the CEPH collection) grandparents.(Jordan, et al. 2012)
- Cases and controls in cohort Utah were recruited from the Department of Dermatology at the University of Utah.(Jordan, et al. 2012)
- Cohort of Toronto were gathered from the University of Toronto and Toronto Western Hospital.(Jordan, et al. 2012)

Reference:

Jordan, C. T., et al.

2012 Rare and common variants in CARD14, encoding an epidermal regulator of NF-kappaB, in psoriasis. Am J Hum Genet 90(5):796-808.

Qin, P., et al.

2014 Variant analysis of CARD14 in a Chinese Han population with psoriasis vulgaris and generalized pustular psoriasis. J Invest Dermatol 134(12):2994-2996.
